# Magnetic resonance brain volumetry biomarkers of CLN2 Batten disease identified with miniswine model

**DOI:** 10.1038/s41598-023-32071-z

**Published:** 2023-03-29

**Authors:** Kevin Knoernschild, Hans J. Johnson, Kimberly E. Schroeder, Vicki J. Swier, Katherine A. White, Takashi S. Sato, Christopher S. Rogers, Jill M. Weimer, Jessica C. Sieren

**Affiliations:** 1 Department of Radiology, University of Iowa, 200 Hawkins Drive cc704 GH, Iowa City, IA 52242 USA; 2 Department of Biomedical Engineering, University of Iowa, Iowa City, IA USA; 3 Holden Comprehensive Cancer Center, University of Iowa, Iowa City, IA USA; 4 Department of Electrical and Computer Engineering, University of Iowa, Iowa City, IA USA; 5 Department of Psychiatry, University of Iowa, Iowa City, IA USA; 6 Pediatrics and Rare Diseases Group, Sanford Research, Sioux Falls, SD USA; 7 Precigen Exemplar, Coralville, IA USA

**Keywords:** Neuroscience, Biomarkers, Diseases, Medical research

## Abstract

Late-infantile neuronal ceroid lipofuscinosis type 2 (CLN2) disease (Batten disease) is a rare pediatric disease, with symptom development leading to clinical diagnosis. Early diagnosis and effective tracking of disease progression are required for treatment. We hypothesize that brain volumetry is valuable in identifying CLN2 disease at an early stage and tracking disease progression in a genetically modified miniswine model. *CLN2*^*R208X/R208X*^ miniswine and wild type controls were evaluated at 12- and 17-months of age, correlating to early and late stages of disease progression. Magnetic resonance imaging (MRI) T1- and T2-weighted data were acquired. Total intercranial, gray matter, cerebrospinal fluid, white matter, caudate, putamen, and ventricle volumes were calculated and expressed as proportions of the intracranial volume. The brain regions were compared between timepoints and cohorts using Gardner-Altman plots, mean differences, and confidence intervals. At an early stage of disease, the total intracranial volume (− 9.06 cm^3^), gray matter (− 4.37% 95 CI − 7.41; − 1.83), caudate (− 0.16%, 95 CI − 0.24; − 0.08) and putamen (− 0.11% 95 CI − 0.23; − 0.02) were all notably smaller in *CLN2*^*R208X/R208X*^ miniswines versus WT, while cerebrospinal fluid was larger (+ 3.42%, 95 CI 2.54; 6.18). As the disease progressed to a later stage, the difference between the gray matter (− 8.27%, 95 CI − 10.1; − 5.56) and cerebrospinal fluid (+ 6.88%, 95 CI 4.31; 8.51) continued to become more pronounced, while others remained stable. MRI brain volumetry in this miniswine model of CLN2 disease is sensitive to early disease detection and longitudinal change monitoring, providing a valuable tool for pre-clinical treatment development and evaluation.

## Introduction

Batten disease, or neuronal ceroid lipofuscinoses, is a group of neurodegenerative diseases in which mutations in 13–14 different genes can cause issues with a lysosome’s capability to recycle or process molecules. All forms of Batten disease show similar symptoms but are caused by different gene mutations^1,2^. The late-infantile neuronal ceroid lipofuscinosis type 2 (CLN2) disease is rare, and usually not diagnosed until the patient is already symptomatic. This disease occurs in children around 2–4 years of age causing early brain degeneration and cognitive decline, in addition to the loss of visual and motor functions, ultimately leading to death^2–4^. These regressions are caused by an enzyme deficiency of tripeptidyl peptidase 1, which causes an inability to remove waste that would normally be metabolized by lysosomes^1^. No cure exists for CLN2 disease, but enzyme replacement therapy protocols have been approved as a method to slow disease progression^5^. For these replacement therapies to be effective, they must be administered as soon as possible. Early disease recognition and initiation of therapy are essential in treating patients with CLN2 disease.

Disease frequency within CLN2 is not well reported across the world population, with sources varying from 0.15 to 9 in 100,000 births^6–8^. This low frequency in the population hinders single-center study recruitment and data collection, making consistent longitudinal data acquisition of humans affected by CLN2 disease difficult. It is particularly challenging to obtain data at a pre-symptomatic stage of the disease. MRI studies have been completed with human subjects with CLN2 disease, but the imaging studies used are at variable points in the disease course due to differences in disease onset, diagnosis, and patient availability^9^. Therefore, there is a need to model CLN2 disease progression from pre- to post-symptomatic stages that is standardized.

One way to analyze disease progression in neurodegenerative diseases is through volumetrics from non-invasive medical imaging, such as MRI, as a measure of cerebral atrophy. Cerebral atrophy is the loss of neurons in the brain, which results in overall brain tissue shrinkage. Batten disease studies in humans have used volumetric analysis to show noteworthy gray matter (GM) and white matter (WM) atrophy over time^9,10^. Additionally, CLN2 disease studies that include volumetric analysis show ventricular expansion and higher overall volumes of cerebrospinal fluid (CSF) compared to a normal brain^10^ An increase in total CSF volume is one potential sign of neurodegenerative progression when observed in combination with intracranial volume (ICV), GM, and WM changes^11,12^.

The purpose of our study is to test our hypothesis that brain volumetry at 12- and 17-months for *CLN2*^*R208X/R208X*^ miniswine differs from that of wild type controls. We hypothesize that degeneration due to *CLN2*^*R208X/R208X*^ will mirror reported brain tissue degeneration in humans with CLN2 disease. Volumetric analysis of specific regional brain degeneration in miniswine affected by *CLN2*^*R208X/R208X*^ can then be used as early biomarkers for disease progression severity in patients and for monitoring pre-clinical therapy response.

## Materials and methods

### Animals

All procedures were approved by the Institutional Animal Care and Use Committees (IACUC) of the University of Iowa and Precigen Exemplar, all methods were performed in accordance with relevant regulations and guidelines, and reported in accordance with ARRIVE guidelines. 23 unique Yucatan miniswine underwent MR imaging over a two-year period. 18 female miniswine were *CLN2*^*R208X/R208X*^confirmed, with processes further described in a characterization paper including behavioral, pathological, and phenotypical analysis^13^. Of these 18 *CLN2*^*R208X/R208X*^ miniswine, 10 animals completed longitudinal imaging procedures at 12-month and 17-month time points. Of the remaining 8 *CLN2*^*R208X/R208X*^ confirmed miniswine, 4 completed imaging at the 12-month time point only, and 4 completed imaging at the 17-month time point only. An additional comparator group of 5 wild type (WT) Yucatan miniswine completed MR imaging at both 12 and 17-month time points. At 12-months of age, *CLN2*^*R208X/R208X*^ miniswine did not have overt symptom development (early disease stage)^13^. By 17-months of age *CLN2*^*R208X/R208X*^ miniswine exhibited late-stage disease symptoms (such as blindness, motor deterioration and/or seizures), mirroring symptoms in *CLN2*^*R208X/R208X*^patients^13^.

Animals were pre- anesthetized with either a combination of telazol (2.2–4.4 mg/kg), ketamine (1.1–2.2 mg/kg) and xylazine (1.1–2.2 mg/kg) or ketamine (22-33 mg/kg) and acepromazine (1.1 mg/kg). Anesthesia was maintained with inhaled isoflurane (1–5%). Animals were intubated with a balloon-cuffed endotracheal tube to maintain the airway and underwent imaging with free-breathing oxygen and isoflurane (~ 2%). If additional respiratory support was needed, mechanical ventilation was administered at a tidal volume of approximately 10 mL/kg and respiratory rate of 18–22 breaths per minute using a Premier SP MRI-compatible veterinary anesthesia ventilator (DRE Veterinary).

### Imaging

MRI data were acquired using a 3 T SIGNA Premier MRI scanner (GE Healthcare) with a medium 16-channel flexible coil (GE Healthcare). Animals were positioned right-side feet first in the MRI scanner. The full miniswine imaging protocol included T1-weighted, T2-weighted, diffusion-weighted imaging, and field map acquisitions. T1-weighted images utilized the BRAVO pulse sequence (TR/TE/TI/flip angle: 7.6/3.3/450 ms/12°; voxel size: 0.7 × 0.7 × 0.7 mm^3^). T2-weighted images utilized the CUBE pulse sequence (TR/TE: 3000/51; voxel size: 0.7 × 0.7 × 0.7 mm^3^). To improve image quality and reduce potential motion artifacts discovered during initial miniswine test scans using longer acquisition times, multiple shorter acquisitions were acquired and then averaged (1–4 acquisitions approximately 6 min each).

### Image pre-processing

Each animals’s T1-weighted and T2-weighted MRI data were visually inspected for motion artifacts before pre-processing. A single reference T1-weighted image was selected for each scanning session and the anterior commissure/posterior commissure aligned. The remaining T1-weighted images for that individual session were rigidly registered to the selected T1-weighted image using the Brain Research: Analysis of Images, Networks and Systems toolkit (BRAINStools)^14,15^. T2-weighted images were registered using rigid and affine transforms to the reference T1w image utilizing the Advanced Normalization Tools toolkit^16^. Registered images were then averaged together based off image type (T1- or T2-weighted respectively) resulting in a single T1-weighted/T2-weighted image pair for each miniswine. These averaged, aligned image pairs then underwent Rician denoising using BRAINStools’ DenoiseImage function.

### Segmentations

Three-dimensional manual segmentations were created for each animal using 3D Slicer’s segmentation editing tool (https://www.slicer.org) and each MRI scan sessions averaged T1w/T2w pairs for boundary reference^17^. Regions of interest included caudate, putamen, lateral ventricles, and the total intracranial volume (ICV) of the skull^10,18^. For consistency, an adapted miniswine version of McRae’s line was used as a reference cutoff point for the intracranial segmentation^19^. Additionally, the Advanced Normalization Tools ATROPOS script was used to create an automated segmentation of the CSF, WM and GM using the default suggested parameters for 3-class tissue segmentation^20^.

### Immunohistochemistry

Brains were histologically examined for classic Batten disease pathology in the somatosensory cortex as this is one of the more commonly examined cortical regions that displays Batten disease pathology^21,22^. Female animals were sacrificed with pentobarbital at 17 months of age, and one hemisphere of the brain was placed into 10% neutral buffered formalin for approximately 3 weeks. The brain was sub-dissected into somatosensory cortex blocks and equilibrated in cryoprotectant solution (30% sucrose in TBSA) at 4 °C. Blocks were serial sectioned (50 µm) on a freezing microtome (Leica) and free-floating sections were used for standard immunohistochemistry^13,23–27^. The following primary antibodies were used: anti-mitochondrial ATP synthase subunit C (Abcam, ab181243; 1:2000). Immunolabeled sections were scanned using an Aperio Versa slide scanner (Leica Biosystems, IL, USA) and at least 3 images were extracted from each region of interest and processed as previously published for total percent area of mitochondrial ATP synthase subunit c (SubC)^13,23,24^.

### Data analysis

Brain region segmentations were used to calculate regional volumes. These volume measurements were expressed as percent of total ICV and used for cohort and longitudinal comparisons between 12- and 17-month *CLN2*^*R208X/R208X*^ and WT pigs. Estimation plots are used in this study to evaluate the differences between groups and time points. A Gardner–Altman plot is an estimation plot that allows transparent visualization of the all the data as a swarm plot, the effect size (difference in the means) and the precision (95% confidence interval)^28,29^. For estimation plots, if the average difference and 95% confidence interval do not cross the horizontal line at zero it indicates a reliable measurement difference between the cohorts. If the 95% confidence interval crosses the horizontal line at zero, as an effect size equal to zero is possible and the measurement difference is unlikely to be reliable. Gardner-Altman plots were created using the DABEST open-source library for R (R, version 4.0.3)^29^. Plots and statistical testing of the SubC data were analyzed in Graphpad Prism 9.0 as specified in the figure legend (****p < 0.0001).

## Results

**Figure 1:**
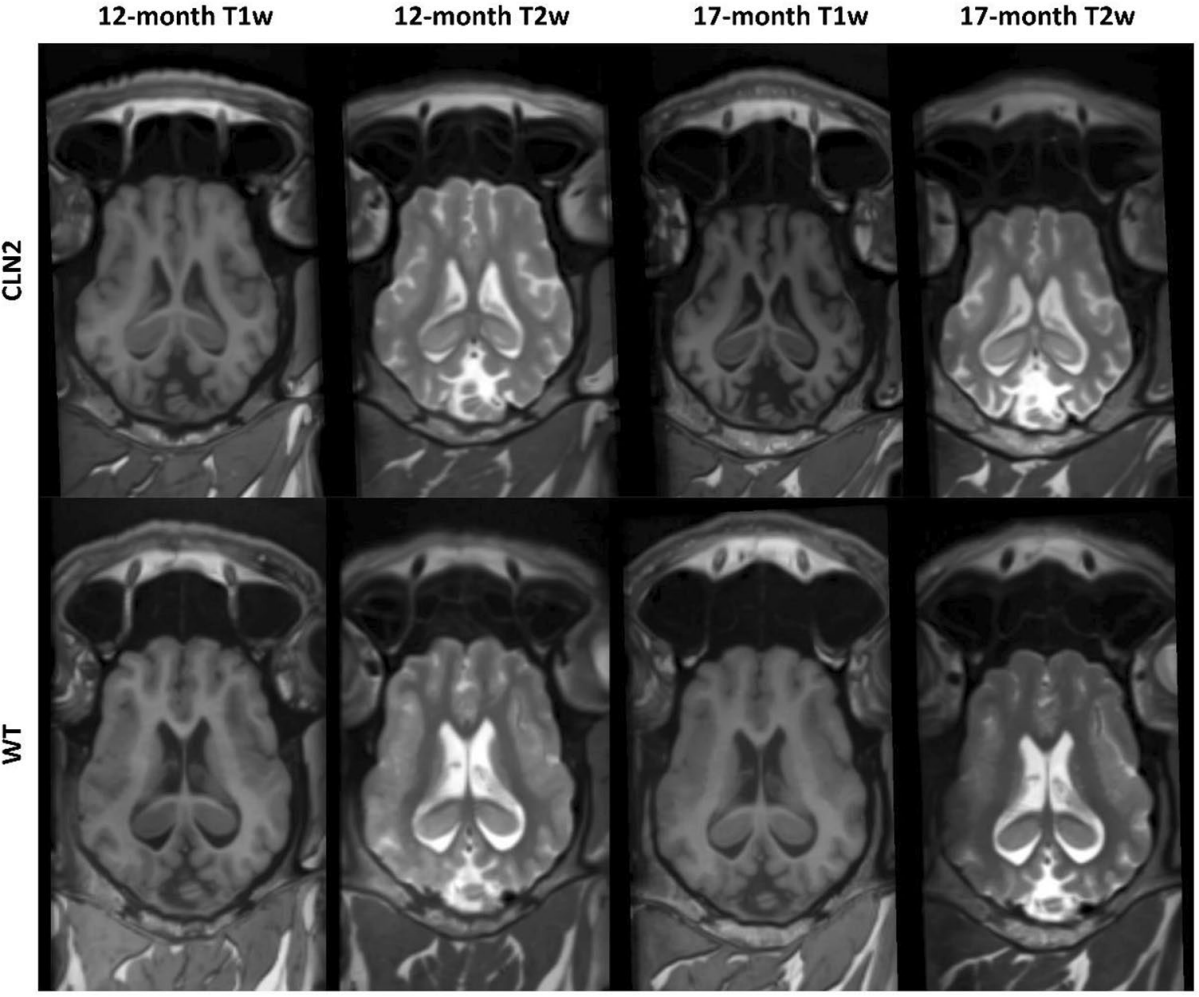
Exemplary T1-weighted (T1w) and T2-weighted (T2w) MRI data from a *CLN2*^*R208X/R208X*^ miniswine at 12-months-of-age (top left) and 17-months (top right), and a wild type (WT) comparator (bottom). Images were aligned using anterior commissure, posterior commissure, and basal pons, so that equivalent axial slices were taken from each time point.

Thirty-eight scans were analyzed, comprised of fourteen 12-month-old *CLN2*^*R208X/R208X*^ miniswine scan sessions, fourteen 17-month-old miniswine scan sessions, five WT 12-month-old miniswine scan sessions, and five WT 17-month-old miniswine scans scan sessions. Ten of the *CLN2*^*R208X/R208X*^ miniswine had scan data available for both 12- and 17-month time points. Representative images of the MRI data for WT and *CLN2*^*R208X/R208X*^ are shown in Fig. 1. All five WT animals had 12- and 17-month time point scans acquired. A single *CLN2*^*R208X/R208X*^ 12-month scan session was left out due to scan quality causing segmentation failure for automated extraction of the GM, WM and CSF. Diffusion-weighted imaging data were collected but included pronounced artifact, caused primarily due to the large, complex sinus structure of the miniswine, hence these data were not quantitatively analyzed in this study.

### Intracranial volume (ICV)

At 12-months, *CLN2*^*R208X/R208X*^ miniswine had a smaller average ICV compared to WT miniswines by 9.06 cm^3^. At 17-months, this ICV volume difference increased, with the *CLN2*^*R208X/R208X*^ cohort having a smaller average ICV by 19.95 cm^3^ compared to 17-month-old WT cohort. The average ICV volume for *CLN2*^*R208X/R208X*^ animals was 100.46 (± 5.53) cm^3^ at 12-months, and 96.22 (± 6.62) cm^3^ at 17-months old (average decrease over time of 4.24 cm^3^). The average ICV volume for 12-month WT animals was 109.52 (± 5.65) cm^3^, and 116.17 (± 4.51) cm^3^ at 17-months (average increase over time of 6.65 cm^3^). A summary of all volume changes is available in Table 1.

**Table 1:** Mean and standard deviation of the volumes for the brain volumetry regions.

Tissue measurement	CLN2 12-month	CLN2 17-month	WT 12-month	WT 17-month
ICV (cm^3^)	100.46 ± 5.53	96.22 ± 6.62	109.52 ± 5.65	116.17 ± 4.51
Caudate (% ICV)	0.72 ± 0.08	0.67 ± 0.08	0.88 ± 0.08	0.80 ± 0.07
Putamen (% ICV)	0.65 ± 0.09	0.59 ± 0.07	0.76 ± 0.11	0.65 ± 0.04
Ventricles (% ICV)	1.72 ± 0.48	2.45 ± 0.37	1.98 ± 1.06	2.24 ± 1.24
Gray Matter (% ICV)	34.71 ± 2.02	29.29 ± 0.96	39.08 ± 2.91	37.56 ± 2.38
White Matter (% ICV)	39.18 ± 3.10	40.16 ± 2.71	38.23 ± 2.98	38.77 ± 1.26
CSF (% ICV)	26.11 ± 2.50	30.55 ± 2.25	22.68 ± 2.47	23.67 ± 2.41

**Figure 2:**
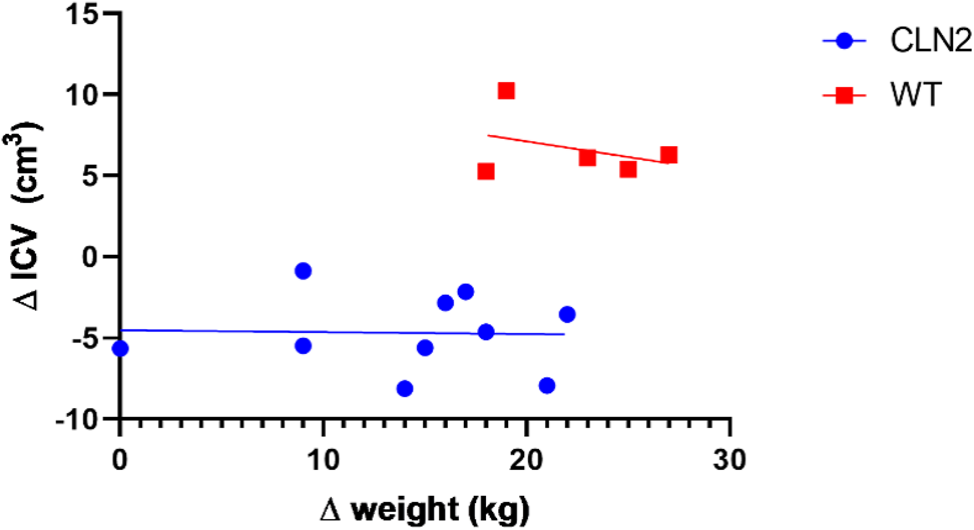
The relationship between weight change (17–12 months) and change in intracranial volume (ICV) (17–12 months) in the *CLN2*^*R208X/R208X*^ miniswine (blue, R^2^ = 0.0011) and WT miniswine (red, R^2^ = 0.13), showing reduction in ICV (volume change range: − 1 to − 8 cm^3^) across *CLN2*^*R208X/R208X*^ miniswine unrelated to the large variation in weight change across the cohort (0 to 22 kg).

We evaluated the relationship between ICV change over time and miniswine weight (Fig. 2). The reduction in ICV between 12- and 17-months-of-age was consistent in *CLN2*^*R208X/R208X*^ miniswine, despite a large diversity in the degree of weight change between the time points (ranging from 0 to 22 kg), with no negative correlation.

### Gray matter (GM)

**Figure 3:**
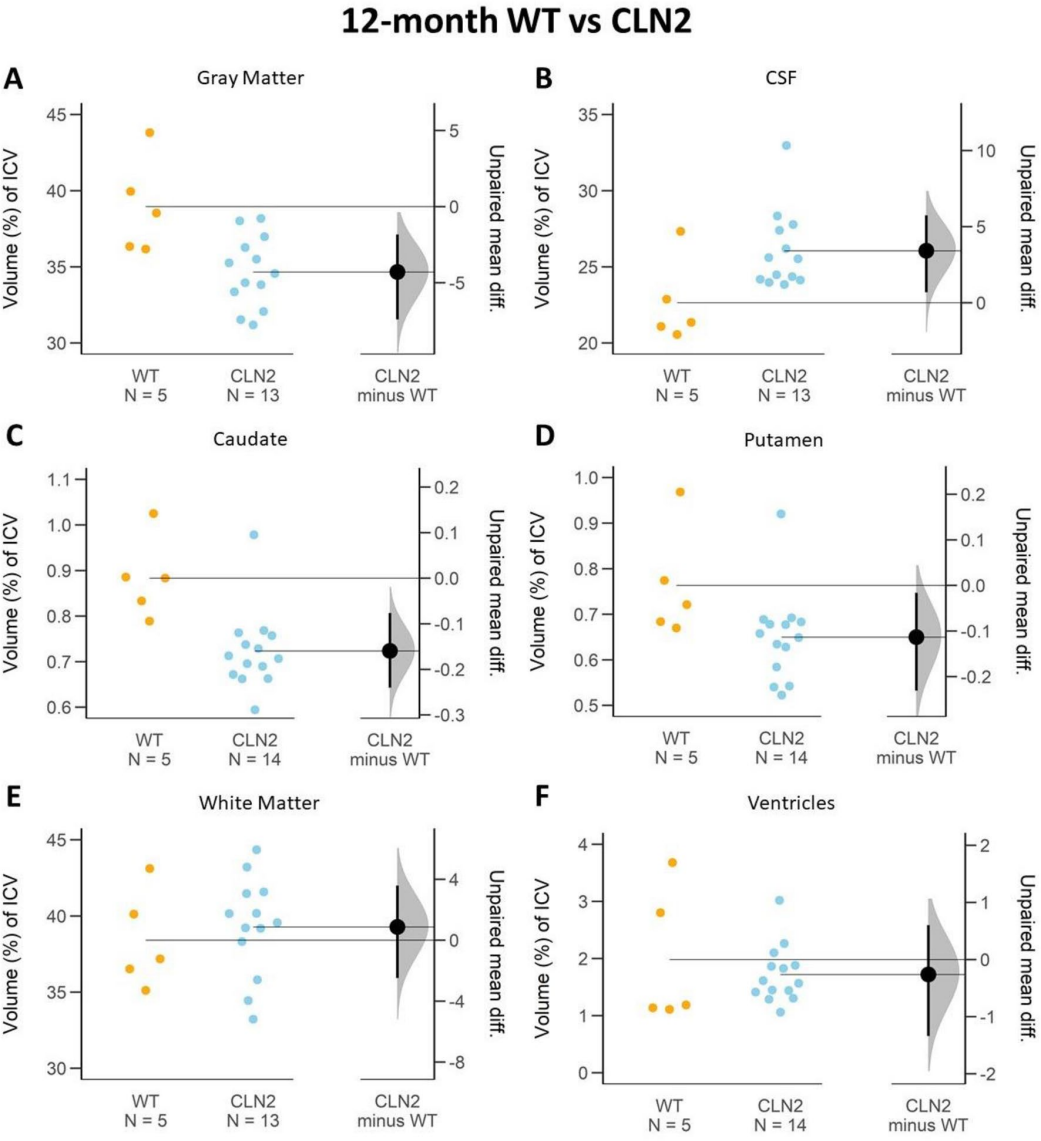
Early disease stage (at 12-months-of-age) ICV proportion comparison of brain regions between WT and *CLN2*^*R208X/R208X*^ cohorts. The average difference and 95% confidence interval (black circle with error bar in *CLN2 minus WT* column) do not cross the horizontal line at zero and indicate reliable measurement difference between the cohorts in gray matter (**A**), CSF (**B**), caudate (**C**), and putamen (**D**). When the average difference and 95% confidence intervals (black circle with error bar in *CLN2 minus WT* column) cross the horizontal line at zero as it does for white matter (**E**) and ventricles (**F**), an effect size equal to zero is possible, and reliable measured difference is unlikely.

**Figure 4:**
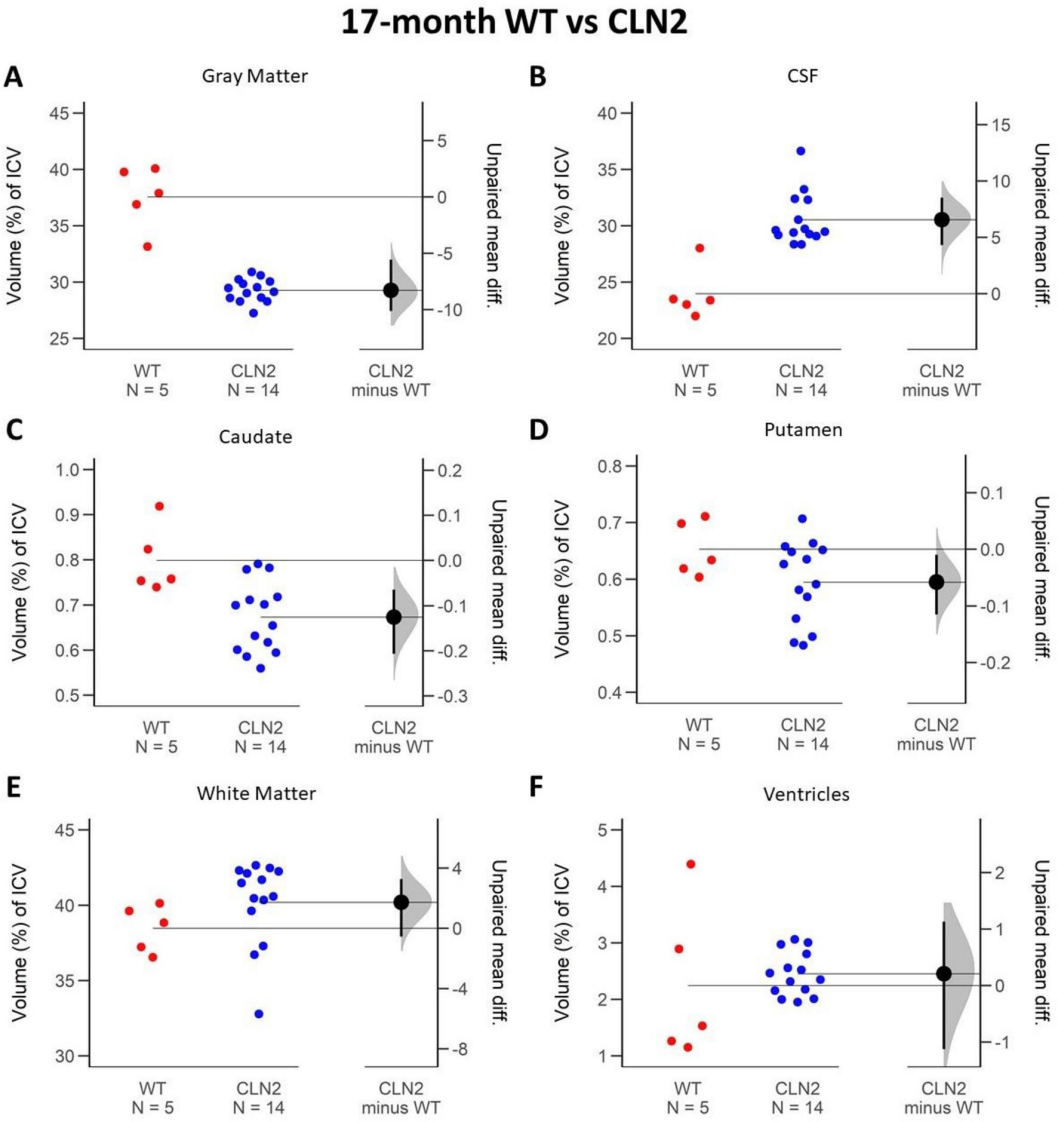
Late disease stage (at 17-months of age) ICV proportion comparison of brain regions between WT and *CLN2*^*R208X/R208X*^ cohorts. The average difference and 95% confidence interval (black circle with error bar in *CLN2 minus WT* column) do not cross the horizontal line at zero and indicate reliable measurement difference between the cohorts in gray matter (**A**), CSF (**B**), caudate (**C**), and putamen (**D**). The average difference and 95% confidence intervals (black circle with error bar in *CLN2 minus WT* column) cross the horizontal line at zero for white matter (**E**) and ventricles (**F**), indicating reliable measured difference is unlikely.

**Figure 5:**
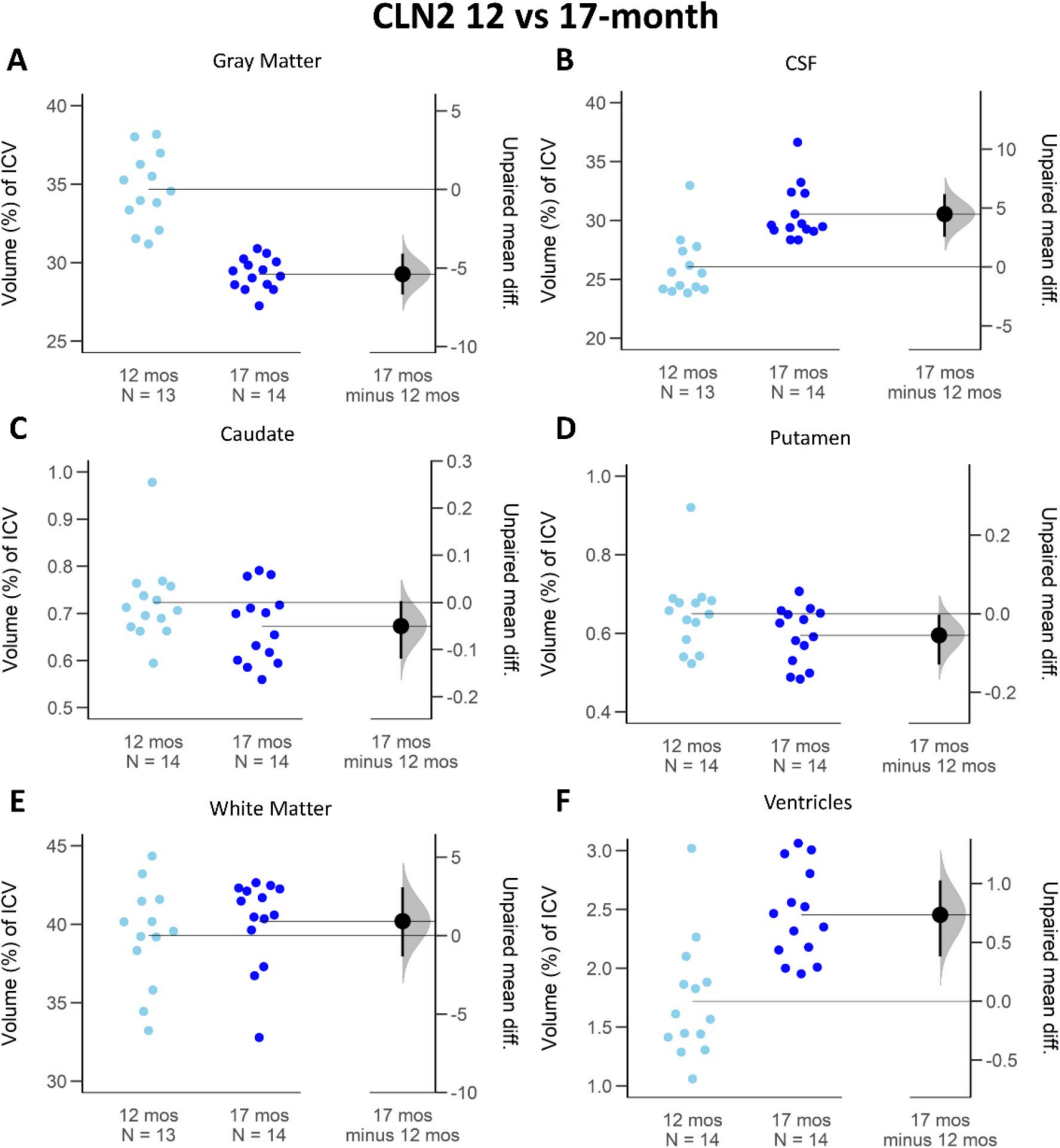
ICV proportion comparison of brain regions between 12- and 17-month-old *CLN2*^*R208X/R208X*^ minipigs. The average difference and 95% confidence interval (black circle with error bar in *17 mos minus 12 mos column*) do not cross the horizontal line at zero and indicates reliable measurement difference between the cohorts in gray matter (**A**), CSF (**B**), putamen (**D**), and ventricles (**F**). When the average difference and 95% confidence intervals (black circle with error bar in *17 mos minus 12 mos* column) cross the horizontal line at zero as it does for caudate (**C**) and white matter (**E**), an effect size equal to zero is possible, and reliable measured difference is unlikely.

**Figure 6:**
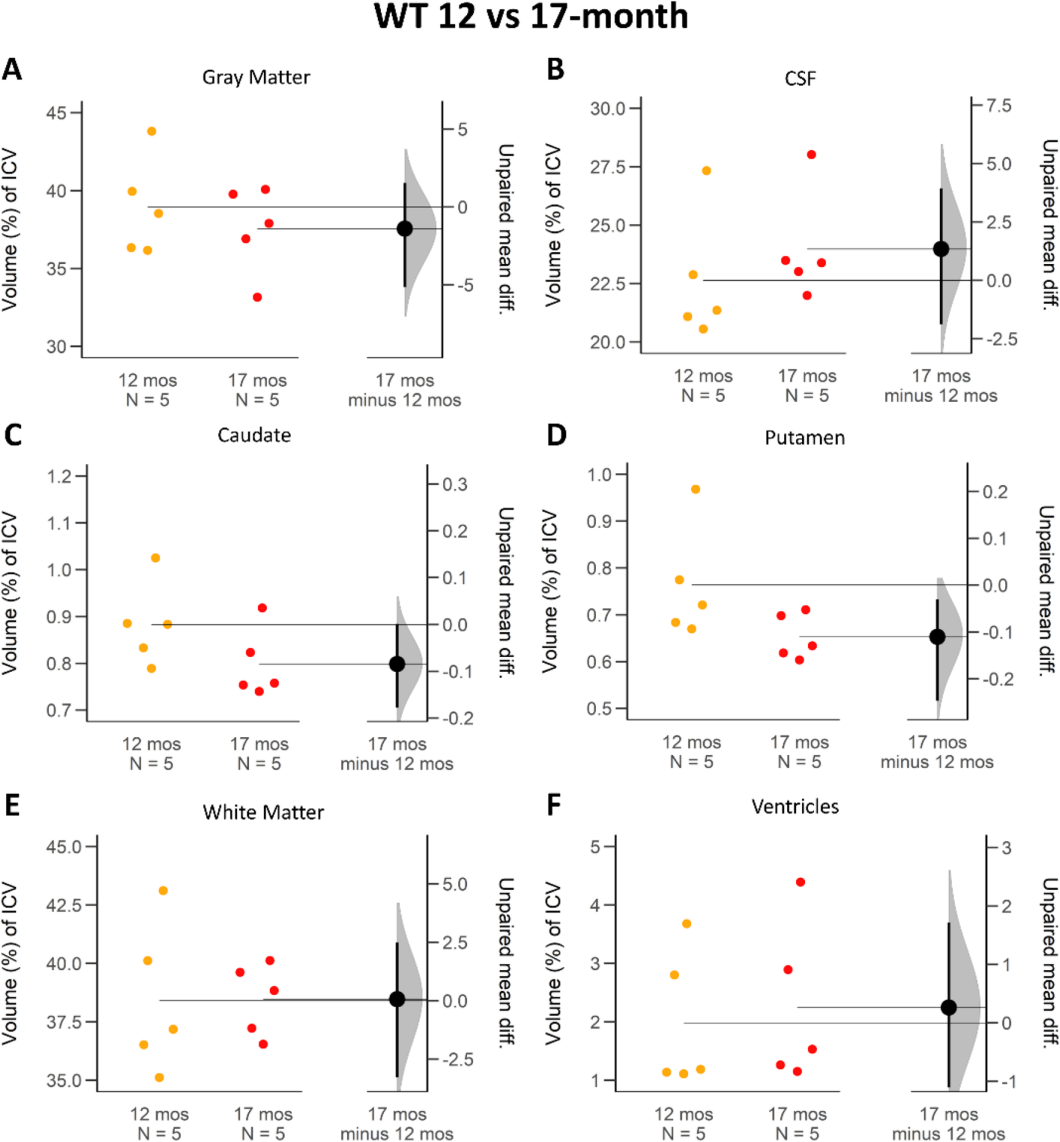
ICV proportion comparison of brain regions between 12- and 17-month-old WT miniswine. The average difference and 95% confidence interval (black circle with error bar in *17 mos minus 12 mos column*) do not cross the horizontal line at zero and indicates reliable measurement difference between the cohorts in the putamen (**D**). When the average difference and 95% confidence intervals (black circle with error bar in *17 mos minus 12 mos* column) cross the horizontal line at zero as it does for gray matter (**A**), CSF (**B**), caudate (**C**), white matter (**E**) and ventricles (**F**), an effect size equal to zero is possible, and reliable measured difference is unlikely.

The largest proportional change in ICV volume between cohorts occurred in the GM ICV proportion measurements. Compared to the WT controls, *CLN2*^*R208X/R208X*^ GM proportion of ICV was smaller by 4.37% at 12 months, and 8.27% at 17 months. The precision of the differences between *CLN2*^*R208X/R208X*^ and WT at both 12- and 17-months are shown in the confidence intervals of Fig. 3A (12-month comparison 95 CI − 7.41; − 1.83) and Fig. 4A (17-month comparison 95 CI − 10.1; − 5.56). As the confidence intervals do not cross zero, there is 95% confidence that the difference between the cohorts is not zero. The *CLN2*^*R208X/R208X*^ cohort showed a longitudinal decrease in GM over time of 5.43% (Fig. 5A, 95 CI − 6.7; − 4.1), while there was no support for a meaningful decrease in the WT (Fig. 6A, 95 CI − 5.15; 1.52).

### Cerebrospinal fluid (CSF)

CSF also showed a large longitudinal ICV proportion change for the *CLN2*^*R208X/R208X*^ animals. CSF proportion of ICV was larger at both 12- and 17-month measurements of *CLN2*^*R208X/R208X*^ affected miniswines compared to WT (12-month: + 3.42%, 17-month: 6.88%). Figure 3B visualizes the confidence interval of a shift in the longitudinal *CLN2*^*R208X/R208X*^ population (95 CI 2.54; 6.18) and Fig. 4B supports the difference between their WT counterparts at 17-months old (95 CI 4.31; 8.51). On average, longitudinal CSF proportion of ICV increased by 4.44% for *CLN2*^*R208X/R208X*^ animals (Fig. 5B, 95 CI 2.54; 6.18). By comparison, the WT had a small increase of 0.98% which was not supported by the confidence interval (Fig. 6B, 95 CI − 1.89; 3.93).

### Caudate and putamen

Caudate and putamen are very small interior structures compared to the ICV, and hence the numerical change longitudinally for the *CLN2*^*R208X/R208X*^ and WT cohorts is also extremely small. *CLN2*^*R208X/R208X*^ caudate ICV proportion compared to the average WT counterpart was smaller at both 12-months (Fig. 3C, − 0.16%, 95 CI − 0.24; − 0.08) and at 17-months (Fig. 4C, − 0.13%, 95 CI − 0.21; − 0.07). *CLN2*^*R208X/R208X*^ putamen ICV proportion at 12 and 17-months was also smaller, than the corresponding WT measurements, at − 0.11% (Fig. 3D, 95 CI − 0.23; − 0.02) and 0.06% (Fig. 4D, 95 CI − 0.12; − 0.01). Over time in the *CLN2*^*R208X/R208X*^ cohort, the average change in ICV proportion was decreased for the putamen (Fig. 5D, − 0.06%, 95 CI − 0.13; − 0.002). This longitudinal decrease similarly occurred in the WT putamen (Fig. 6D, − 0.11%, 95 CI − 0.25; − 0.03). However, for the longitudinal change in caudate ICV proportion there was not a reliable difference in either cohort (Figs. 5C, 6C).

### White matter (WM)

WM proportional ICV volumes were relatively stable between the *CLN2*^*R208X/R208X*^ and WT cohorts. Compared with the WT cohort, the *CLN2*^*R208X/R208X*^ miniswine had non-significant, slightly higher percentage of ICV occupied by WM at 12-months (Fig. 3E: + 0.95%, 95 CI − 2.48; 3.58) and at 17-months (Fig. 4E: + 1.39%, 95 CI − 0.55; 3.27). Longitudinal changes between 12- and 17-months were not supported by the confidence interval to have a reliable difference for either cohort: the *CLN2*^*R208X/R208X*^ difference of + 0.98% (95 CI − 1.34; 3.06) (Fig. 5E) and WT difference of + 0.54% (95 CI − 3.28; 2.48) (Fig. 6E).

### Ventricles

Average proportion of ICV for lateral ventricles increased significantly over time for *CLN2*^*R208X/R208X*^ animals by 0.73% (Fig. 5F, 95 CI 0.38; 1.03) compared to no meaningful change in WT animals (Fig. 6F, 0.26% 95 CI − 1.11; 1.71). At 12-months, *CLN2*^*R208X/R208X*^ miniswine had a lateral ventricle average ICV proportion of 1.72 (± 0.48)% compared to the WT measure of 1.98 (± 1.06)%. Figures 3F and 4F illustrate that comparing the *CLN2*^*R208X/R208X*^ cohort to the WT cohort, there was little difference between the two in the proportion of ICV for lateral ventricles.

### Immunohistochemistry

**Figure 7:**
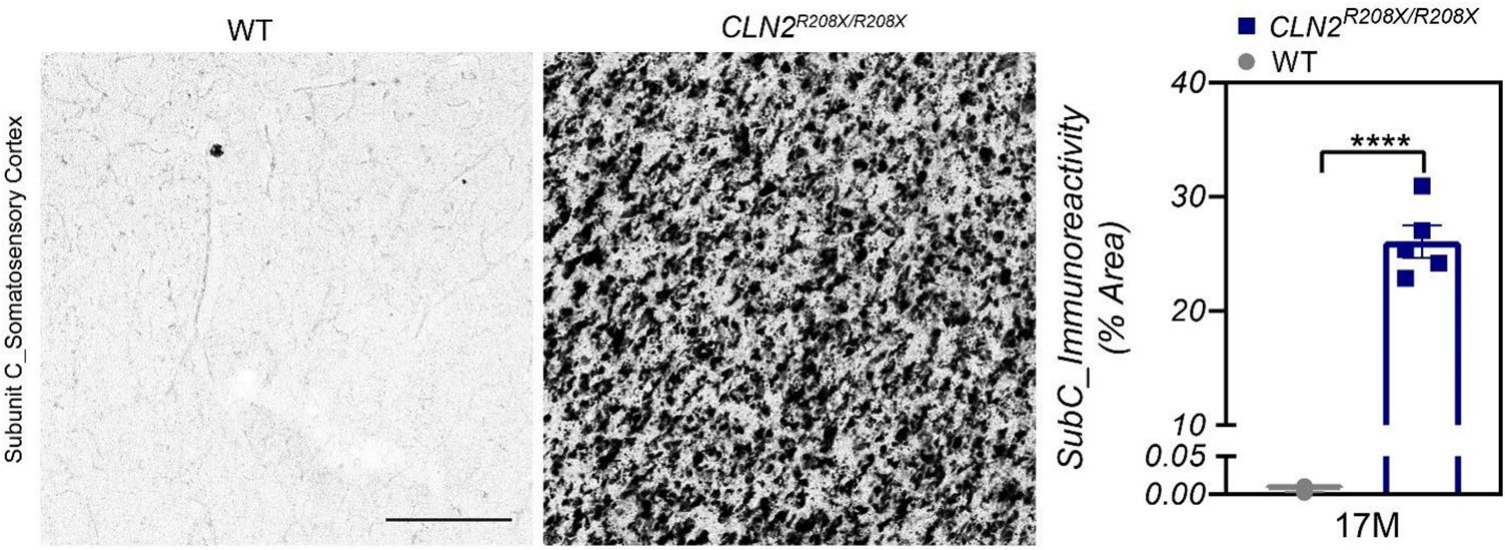
Mitochondrial ATP synthase subunit c accumulates in the somatosensory cortex of *CLN2*^*R208X/R208X*^ animals. Subunit c accumulation shown in the somatosensory cortex at 17-months-of-age. Mean ± SEM, Nested t-test. ****p < 0.0001. Scale bar = 200 µm.

At 17-months-of-age, SubC significantly accumulates in the somatosensory cortex of *CLN2*^*R208X/R208X*^ animals compared to age matched, WT counterparts (Fig. 7). While correlation between SubC and MRI brain volumetry did not reach statistical significance for this small sub-cohort, there was a weak trend of lower ICV and GM volumes at higher SubC values for *CLN2*^*R208X/R208X*^ miniswine at 17-months-of-age (Supplementary Fig. 1).

## Discussion

This study identified MRI biomarkers of early disease and longitudinal change in a cohort of *CLN2*^*R208X/R208X*^ versus WT miniswines. At an early stage of disease progression (12-months-of-age in the miniswine model), we found meaningful differences in the ICV proportional volumes of GM (reduced in *CLN2*^*R208X/R208X*^), caudate (reduced in *CLN2*^*R208X/R208X*^), putamen (reduced in *CLN2*^*R208X/R208X*^) and CSF (elevated in *CLN2*^*R208X/R208X*^) between *CLN2*^*R208X/R208X*^ and WT cohorts. As the diseased progressed to a late stage (17-months-of-age in the miniswine model), the difference in the ICV proportional volumes of GM and CSF became more pronounced, while the other measurement differences remained relatively consistent to the 12-month relationship. This indicates that evaluation of MRI derived brain volumes could be utilized to monitor treatment response in preclinical miniswine studies.

Characterization of the *CLN2*^*R208X/R208X*^ miniswine model used for this study has recently been reported by Swier et al.^13^ incorporating behavioral, pathological, and visual testing regularly from 3-months to 18-months-of-age. This study demonstrated the age of onset of phenotypes for female *CLN2*^*R208X/R208X*^ miniswine including overt vision deficits at a mean age of 15-months, motor coordination loss at a mean age of 16-months, and early death at a mean age of 17.5-months. At 12 months-of-age, reversal learning deficits were observed in *CLN2*^*R208X/R208X*^ females during t-maze testing^13^, similar to that observed in other Batten disease large animal models *(TPP1*^*−/−*^ dog and *CLN5* sheep models)^30–32^, as animals approached end-stage, reversal deficits increased. Accumulation of SubC in the somatosensory cortex, consistent with the findings from Swier et al.^13^, are presented for a subset of the miniswine that underwent MRI analysis as a connection to these characterization studies. Correlation between SubC measures and MRI brain volumetry did not reach statistical significance in this study, potentially due to the small sample size for which ex-vivo tissue samples were available.

Miniswine present a valuable translational research tool for exploring and optimizing medical image acquisition and analysis, understanding disease etiology, and exploring innovative treatment options. Genetically modified miniswine have been used for advancing a broad range of diseases including cystic fibrosis, cancer, neurofibromatosis, and others^33–47^. For neurological studies, the brain morphology development and gray to white matter ratios in miniswine are similar to humans. Porcine models have been used to study neurodegenerative diseases, including Alzheimer’s and traumatic brain injury^48,49^. Genetically modified miniswine can have standardized brain image acquisition times at pre-determined longitudinal timepoints, creating a more consistent representation of *CLN2*^*R208X/R208X*^ disease progression which is challenging to achieve in humans for rare pediatric diseases. Beyond the miniswine, Batten disease imaging studies have been completed in other large animal models such as sheep (CLN5 and CLN6)^50^, and non-human primates (CLN7), as well as CLN2 dachshund studies that focused on multifocal retinopathy and extraneuronal pathology^39,51,52^.

Expression of brain volumetry as a proportion of total ICV is commonly used in neurological disease studies to compensate between-subject variation in head size at both juvenile and elderly stage disease progressions^53,54^. In this miniswine model, ICV volumes showed a longitudinal decrease across the *CLN2*^*R208X/R208X*^ cohort, as opposed to ICV longitudinal volume increase for the WT cohort. The average ICV volumetric difference at the 17-month time point was larger between the *CLN2*^*R208X/R208X*^ and WT cohorts than at 12-months. This serves as a baseline identifier that is consistent with a recent longitudinal NCL sheep study, which also showed a longitudinal decrease in ICV for CLN5 and CLN6 affected sheep as compared to a control cohort that showed an ICV increase over time^50,55^. Russell et al. hypothesized this ICV reduction could be caused by ventricular shunting^50^. Ventricular shunting, or a widening in the CSF fluid draining pathways in the brain, can cause decreased pressure of the brain and CSF against the skull. This may result in a thickening of the skull to balance out the pressure differential of the affected subjects and a decreased ICV. Our results indicate that while some *CLN2*^*R208X/R208X*^ miniswine failed to thrive as indicated by low weight gain over a five-month period, there was no correlation between change in ICV volume and weight change (Fig. 2).

GM ICV proportional measurements at both 12- and 17-months showed the largest longitudinal proportion change, which is in line with other neurodegenerative diseases effecting motor control such as Alzheimer’s and Huntington’s disease^56,57^. The longitudinal progression trend for GM ICV proportion follows that of a human study of CLN2 disease that showed a decrease in GM is a significant marker for disease progression, with the highest decrease found in the supratentorial region of the brain^9,10,58^. The longitudinal increase in CSF associated with CLN2 disease progression, as was found in our *CLN2*^*R208X/R208X*^ miniswine and human patients^10,59^, supports this miniswine model as a translatable model. In our study, caudate and putamen proportion of ICV in *CLN2*^*R208X/R208X*^ miniswine were smaller than WT counterparts. This finding is supported by similar findings by Löbel et al. in a human patient study that examined segmentation of basal ganglia and thalami in CLN2^10^. The increase in ventricle proportion in the *CLN2*^*R208X/R208X*^ miniswine mimics that reported in a previous human study of CLN2 disease^10^. The slight change in WT ventricular volume is similar to patient studies assessing lateral ventricle volume trajectories in non-diseased subjects^60^.

This study includes some limitations. The research cost associated with large animal studies is higher than that of small animal studies or human studies utilizing clinically acquired medical imaging data, due to the animal purchase cost, housing per-diems, and expense of time on research-dedicated MRI systems. Due to cost-related restrictions the cohort selection for *CLN2*^*R208X/R208X*^ and WT miniswine was limited to female animals only as uncastrated male miniswine present additional challenges for longitudinal imaging and housing. Additionally, to maximize the number of *CLN2*^*R208X/R208X*^ miniswine in the study, the WT cohort was smaller. There was more variability in the WT raw and ICV proportional ventricle volume compared to the *CLN2*^*R208X/R208X*^ cohort, and two WT miniswine had much larger ventricular volumes compared to the other three. The WT cohort pedigree was examined and no parental/genealogical pattern for the phenotype was found. We found no meaningful measurement difference in the ICV proportional ventricle volumes between the WT and *CLN2*^*R208X/R208X*^ cohort but due to the diversity in WT volumes, further study of the ventricles in a larger, future cohort will be required.

This dataset provides a valuable resource for future analysis. In this study diffusion weighted image data were collected but not analyzed due to significant artifact, however, future efforts in post-acquisition artifact correction could permit investigation of this additional data. Recent work by Norris et al., has generated an MRI brain template for male Yucatan miniswine^61^. The dataset and segmentations produced in our study could be used in the future to optimize parameterization and validate precision of the atlas for segmentation. In addition, post validation the atlas may be used to expand the brain segmentation regions for assessment in this female CLN2/WT cohort.

## Conclusion

The purpose of our study was to examine brain volumetry at early (12-months) and late (17-months) stages of disease development in *CLN2*^*R208X/R208X*^ miniswine and illustrate notable differences compared to that of wild type miniswine. We found valuable early disease MRI biomarkers in the ICV, GM, caudate and putamen (all reduced), along with the CSF (increased) in *CLN2*^*R208X/R208X*^ compared to WT. For tracking progressive disease, GM and CSF differences continued to become more pronounced over time. We found supporting evidence from the literature that the miniswine findings recapitulated observations in patients with CLN2 disease, thus supporting the utilization of MRI brain volumetry in *CLN2*^*R208X/R208X*^ miniswine as a valuable pre-clinical tool set for exploring disease etiology and treatment development.

## Supplementary Information

Supplementary Figure 1.

## Data Availability

The datasets generated during and/or analyzed during the current study are available from the corresponding author on reasonable request.

## Abbreviations

CLN2: Late-infantile neuronal ceroid lipofuscinosis type 2
MRI: Magnetic resonance imaging
GM: Gray matter
WM: White matter
CSF: Cerebrospinal fluid
ICV: Intracranial volume
WT: Wild type (control)
CI: Confidence interval
SubC: Mictochondrial ATP synthase subunit c

## References

[1] Mole SE, Cotman SL (2015). Genetics of the neuronal ceroid lipofuscinoses (batten disease). Biochim. Biophys. Acta.

[2] Mole S, Williams R, Goebel H (2012). The Neuronal Ceroid Lipofuscinoses (Batten Disease).

[3] Nickel M, Simonati A, Jacoby D, Lezius S, Kilian D, Van de Graaf B, Pagovich OE, Kosofsky B, Yohay K, Downs M (2018). Disease characteristics and progression in patients with late-infantile neuronal ceroid lipofuscinosis type 2 (CLN2) disease: An observational cohort study. Lancet Child Adolesc. Health.

[4] Kovacs KD, Patel S, Orlin A, Kim K, Van Everen S, Conner T, Sondhi D, Kaminsky SM, D'Amico DJ, Crystal RG (2020). Symmetric age association of retinal degeneration in patients with CLN2-associated batten disease. Ophthalmol. Retina.

[5] Specchio N, Pietrafusa N, Trivisano M (2020). Changing times for CLN2 disease: The era of enzyme replacement therapy. Ther. Clin. Risk Manag..

[6] Claussen M, Heim P, Knispel J, Goebel HH, Kohlschütter A (1992). Incidence of neuronal ceroid-lipofuscinoses in West Germany: Variation of a method for studying autosomal recessive disorders. Am. J. Med. Genet..

[7] Moore SJ, Buckley DJ, MacMillan A, Marshall HD, Steele L, Ray PN, Nawaz Z, Baskin B, Frecker M, Carr SM (2008). The clinical and genetic epidemiology of neuronal ceroid lipofuscinosis in Newfoundland. Clin. Genet..

[8] Uvebrant P, Hagberg B (1997). Neuronal ceroid lipofuscinoses in scandinavia: Epidemiology and clinical pictures. Neuropediatrics.

[9] Dyke JP, Sondhi D, Voss HU, Yohay K, Hollmann C, Mancenido D, Kaminsky SM, Heier LA, Rudser KD, Kosofsky B (2016). Brain region-specific degeneration with disease progression in late infantile neuronal ceroid lipofuscinosis (CLN2 disease). Am. J. Neuroradiol..

[10] Löbel U, Sedlacik J, Nickel M, Lezius S, Fiehler J, Nestrasil I, Kohlschütter A, Schulz A (2016). Volumetric description of brain atrophy in neuronal ceroid lipofuscinosis 2: Supratentorial gray matter shows uniform disease progression. Am. J. Neuroradiol..

[11] De Vis JB, Zwanenburg JJ, van der Kleij LA, Spijkerman JM, Biessels GJ, Hendrikse J, Petersen ET (2016). Cerebrospinal fluid volumetric MRI mapping as a simple measurement for evaluating brain atrophy. Eur. Radiol..

[12] Wardlaw JM, Smith EE, Biessels GJ, Cordonnier C, Fazekas F, Frayne R, Lindley RI, O'Brien JT, Barkhof F, Benavente OR (2013). Neuroimaging standards for research into small vessel disease and its contribution to ageing and neurodegeneration. Lancet Neurol..

[13] Swier VJ, White KA, Johnson TB, Sieren JC, Johnson HJ, Knoernschild K, Wang X, Rohret FA, Rogers CS, Pearce DA (2022). A novel porcine model of CLN2 batten disease that recapitulates patient phenotypes. Neurotherapeutics.

[14] *BRAINSFit: Mutual Information Registrations of Whole-Brain 3D Images, Using the Insight Toolkit* (2007).

[15] Kim REY, Nopoulos P, Paulsen J, Johnson H, Kim REY (2016). Efficient and extensible workflow: Reliable whole brain segmentation for large-scale, multi-center longitudinal human MRI analysis using high performance/throughput computing resources. Clinical Image-Based Procedures Translational Research in Medical Imaging: 2016.

[16] Avants BB, Tustison N, Song G (2009). Advanced normalization tools (ANTS). Insight J..

[17] Fedorov A, Beichel R, Kalpathy-Cramer J, Finet J, Fillion-Robin J-C, Pujol S, Bauer C, Jennings D, Fennessy F, Sonka M (2012). 3D Slicer as an image computing platform for the quantitative imaging network. Magn. Reson. Imaging.

[18] Schubert R, Frank F, Nagelmann N, Liebsch L, Schuldenzucker V, Schramke S, Wirsig M, Johnson H, Kim EY, Ott S (2016). Neuroimaging of a minipig model of Huntington's disease: Feasibility of volumetric, diffusion-weighted and spectroscopic assessments. J. Neurosci. Methods.

[19] McRae DL, Barnum AS (1953). Occipitalization of the atlas. Am. J. Roentgenol..

[20] Avants BB, Tustison NJ, Wu J, Cook PA, Gee JC (2011). An open source multivariate framework for n-tissue segmentation with evaluation on public data. Neuroinformatics.

[21] Sleat DE, Wiseman JA, El-Banna M, Kim KH, Mao Q, Price S, Macauley SL, Sidman RL, Shen MM, Zhao Q (2004). A mouse model of classical late-infantile neuronal ceroid lipofuscinosis based on targeted disruption of the CLN2 gene results in a loss of tripeptidyl-peptidase I activity and progressive neurodegeneration. J. Neurosci..

[22] Kielar C, Maddox L, Bible E, Pontikis CC, Macauley SL, Griffey MA, Wong M, Sands MS, Cooper JD (2007). Successive neuron loss in the thalamus and cortex in a mouse model of infantile neuronal ceroid lipofuscinosis. Neurobiol. Dis..

[23] Johnson TB, Langin LM, Zhao J, Weimer JM, Pearce DA, Kovács AD (2019). Changes in motor behavior, neuropathology, and gut microbiota of a Batten disease mouse model following administration of acidified drinking water. Sci. Rep..

[24] Langin L, Johnson TB, Kovács AD, Pearce DA, Weimer JM (2020). A tailored Cln 3(Q352X) mouse model for testing therapeutic interventions in CLN3 Batten disease. Sci. Rep..

[25] Poppens MJ, Cain JT, Johnson TB, White KA, Davis SS, Laufmann R, Kloth AD, Weimer JM (2019). Tracking sex-dependent differences in a mouse model of CLN6-Batten disease. Orphanet J. Rare Dis..

[26] Swier VJ, White KA, Meyerholz DK, Chefdeville A, Khanna R, Sieren JC, Quelle DE, Weimer JM (2020). Validating indicators of CNS disorders in a swine model of neurological disease. PLoS ONE.

[27] Johnson TB, White KA, Brudvig JJ, Cain JT, Langin L, Pratt MA, Booth CD, Timm DJ, Davis SS, Meyerink B (2021). AAV9 gene therapy increases lifespan and treats pathological and behavioral abnormalities in a mouse model of CLN8-batten disease. Mol. Ther..

[28] Gardner MJ, Altman DG (1986). Confidence intervals rather than P values: Estimation rather than hypothesis testing. Br. Med. J..

[29] Ho J, Tumkaya T, Aryal S, Choi H, Claridge-Chang A (2019). Moving beyond P values: Data analysis with estimation graphics. Nat. Methods.

[30] Katz ML, Coates JR, Sibigtroth CM, Taylor JD, Carpentier M, Young WM, Wininger FA, Kennedy D, Vuillemenot BR, O'Neill CA (2014). Enzyme replacement therapy attenuates disease progression in a canine model of late-infantile neuronal ceroid lipofuscinosis (CLN2 disease). J. Neurosci. Res..

[31] Katz ML, Tecedor L, Chen Y, Williamson BG, Lysenko E, Wininger FA, Young WM, Johnson GC, Whiting RE, Coates JR (2015). AAV gene transfer delays disease onset in a TPP1-deficient canine model of the late infantile form of Batten disease. Sci. Transl. Med..

[32] Mitchell NL, Russell KN, Wellby MP, Wicky HE, Schoderboeck L, Barrell GK, Melzer TR, Gray SJ, Hughes SM, Palmer DN (2018). Longitudinal in vivo monitoring of the CNS demonstrates the efficacy of gene therapy in a sheep model of CLN5 batten disease. Mol. Ther..

[33] Cooney AL, Thornell IM, Singh BK, Shah VS, Stoltz DA, McCray PB, Zabner J, Sinn PL (2019). A novel AAV-mediated gene delivery system corrects CFTR function in pigs. Am. J. Respir. Cell Mol. Biol..

[34] Pino-Argumedo MI, Fischer AJ, Hilkin BM, Gansemer ND, Allen PD, Hoffman EA, Stoltz DA, Welsh MJ, Abou Alaiwa MH (2022). Elastic mucus strands impair mucociliary clearance in cystic fibrosis pigs. Proc. Natl. Acad. Sci. U.S.A..

[35] Welsh MJ, Rogers CS, Stoltz DA, Meyerholz DK, Prather RS (2009). Development of a porcine model of cystic fibrosis. Trans. Am. Clin. Climatol. Assoc..

[36] Hryhorowicz M, Lipiński D, Hryhorowicz S, Nowak-Terpiłowska A, Ryczek N, Zeyland J (2020). Application of genetically engineered pigs in biomedical research. Genes.

[37] Zettler S, Renner S, Kemter E, Hinrichs A, Klymiuk N, Backman M, Riedel EO, Mueller C, Streckel E, Braun-Reichhart C (2020). A decade of experience with genetically tailored pig models for diabetes and metabolic research. Anim. Reprod..

[38] Zhang J, Khazalwa EM, Abkallo HM, Zhou Y, Nie X, Ruan J, Zhao C, Wang J, Xu J, Li X (2021). The advancements, challenges, and future implications of the CRISPR/Cas9 system in swine research. J. Genet. Genom..

[39] McBride JL, Neuringer M, Ferguson B, Kohama SG, Tagge IJ, Zweig RC, Renner LM, McGill TJ, Stoddard J, Peterson S (2018). Discovery of a CLN7 model of Batten disease in non-human primates. Neurobiol. Dis..

[40] Sieren JC, Meyerholz DK, Wang XJ, Davis BT, Newell JD, Hammond E, Rohret JA, Rohret FA, Struzynski JT, Goeken JA (2014). Development and translational imaging of a TP53 porcine tumorigenesis model. J. Clin. Investig..

[41] Hendricks-Wenger A, Nagai-Singer MA, Uh K, Vlaisavljevich E, Lee K, Allen IC (2022). Employing novel porcine models of subcutaneous pancreatic cancer to evaluate oncological therapies. Methods Mol. Biol..

[42] Boas FE, Nurili F, Bendet A, Cheleuitte-Nieves C, Basturk O, Askan G, Michel AO, Monette S, Ziv E, Sofocleous CT (2020). Induction and characterization of pancreatic cancer in a transgenic pig model. PLoS ONE.

[43] Ehrenfeld M, Schrade A, Flisikowska T, Perl M, Hirsch ND, Sichler A, Geyer L, Flisikowski K, Wilhelm D, Schober SJ (2022). Tumor targeting with bacterial shiga toxin B subunit in genetic porcine models for colorectal cancer and osteosarcoma. Mol. Cancer Ther..

[44] Rubinstein CD, McLean DT, Lehman BP, Meudt JJ, Schomberg DT, Krentz KJ, Reichert JL, Meyer MB, Adams M, Konsitzke CM (2021). Assessment of mosaicism and detection of cryptic alleles in CRISPR/Cas9-engineered neurofibromatosis type 1 and TP53 mutant porcine models reveals overlooked challenges in precision modeling of human diseases. Front. Genet..

[45] White KA, Swier VJ, Cain JT, Kohlmeyer JL, Meyerholz DK, Tanas MR, Uthoff J, Hammond E, Li H, Rohret FA (2018). A porcine model of neurofibromatosis type 1 that mimics the human disease. JCI Insight.

[46] Uthoff J, Larson J, Sato TS, Hammond E, Schroeder KE, Rohret F, Rogers CS, Quelle DE, Darbro BW, Khanna R (2020). Longitudinal phenotype development in a minipig model of neurofibromatosis type 1. Sci. Rep..

[47] Osum SH, Watson AL, Largaespada DA (2021). Spontaneous and engineered large animal models of neurofibromatosis type 1. Int. J. Mol. Sci..

[48] Hoffe B, Holahan MR (2019). The use of pigs as a translational model for studying neurodegenerative diseases. Front. Physiol..

[49] Søndergaard LV, Herskin MS, Ladewig J, Holm IE, Dagnæs-Hansen F (2012). Effect of genetic homogeneity on behavioural variability in an object recognition test in cloned Göttingen minipigs. Appl. Anim. Behav. Sci..

[50] Russell KN, Mitchell NL, Anderson NG, Bunt CR, Wellby MP, Melzer TR, Barrell GK, Palmer DN (2018). Computed tomography provides enhanced techniques for longitudinal monitoring of progressive intracranial volume loss associated with regional neurodegeneration in ovine neuronal ceroid lipofuscinoses. Brain Behav..

[51] Katz ML, Johnson GC, Leach SB, Williamson BG, Coates JR, Whiting REH, Vansteenkiste DP, Whitney MS (2017). Extraneuronal pathology in a canine model of CLN2 neuronal ceroid lipofuscinosis after intracerebroventricular gene therapy that delays neurological disease progression. Gene Therapy.

[52] Whiting RE, Pearce JW, Castaner LJ, Jensen CA, Katz RJ, Gilliam DH, Katz ML (2015). Multifocal retinopathy in Dachshunds with CLN2 neuronal ceroid lipofuscinosis. Exp. Eye Res..

[53] Nordenskjöld R, Malmberg F, Larsson E-M, Simmons A, Brooks SJ, Lind L, Ahlström H, Johansson L, Kullberg J (2013). Intracranial volume estimated with commonly used methods could introduce bias in studies including brain volume measurements. Neuroimage.

[54] Tereshchenko A, Magnotta V, Epping E, Mathews K, Espe-Pfeifer P, Martin E, Dawson J, Duan W, Nopoulos P (2019). Brain structure in juvenile-onset Huntington disease. Neurology.

[55] Caspi Y, Brouwer RM, Schnack HG, van de Nieuwenhuijzen ME, Cahn W, Kahn RS, Niessen WJ, van der Lugt A, Pol HH (2020). Changes in the intracranial volume from early adulthood to the sixth decade of life: A longitudinal study. Neuroimage.

[56] Anderson VM, Schott JM, Bartlett JW, Leung KK, Miller DH, Fox NC (2012). Gray matter atrophy rate as a marker of disease progression in AD. Neurobiol. Aging.

[57] Coppen EM, Jacobs M, van den Berg-Huysmans AA, van der Grond J, Roos RAC (2018). Grey matter volume loss is associated with specific clinical motor signs in Huntington's disease. Parkinsonism Relat. Disord..

[58] Schulz A, Ajayi T, Specchio N, de Los RE, Gissen P, Ballon D, Dyke JP, Cahan H, Slasor P, Jacoby D (2018). Study of intraventricular cerliponase alfa for CLN2 disease. N. Engl. J. Med..

[59] Dyke JP, Sondhi D, Voss HU, Shungu DC, Mao X, Yohay K, Worgall S, Hackett NR, Hollmann C, Yeotsas ME (2013). Assessment of disease severity in late infantile neuronal ceroid lipofuscinosis using multiparametric MR imaging. Am. J. Neuroradiol..

[60] Lundervold AJ, Vik A, Lundervold A (2019). Lateral ventricle volume trajectories predict response inhibition in older age—A longitudinal brain imaging and machine learning approach. PLoS ONE.

[61] Norris C, Lisinski J, McNeil E, VanMeter JW, VandeVord P, LaConte SM (2021). MRI brain templates of the male Yucatan minipig. Neuroimage.

