# Supplementary material for: Magnetic resonance brain volumetry biomarkers of CLN2 Batten disease identified with miniswine model
Source: Sci Rep. 2023 Mar 29;13:5146. doi: 10.1038/s41598-023-32071-z (PMC10060411; doi:10.1038/s41598-023-32071-z)
Supplement: Supplementary file 1 — Supplementary Figure 1. [file 41598_2023_32071_MOESM1_ESM.docx]

**Supplementary Figure**


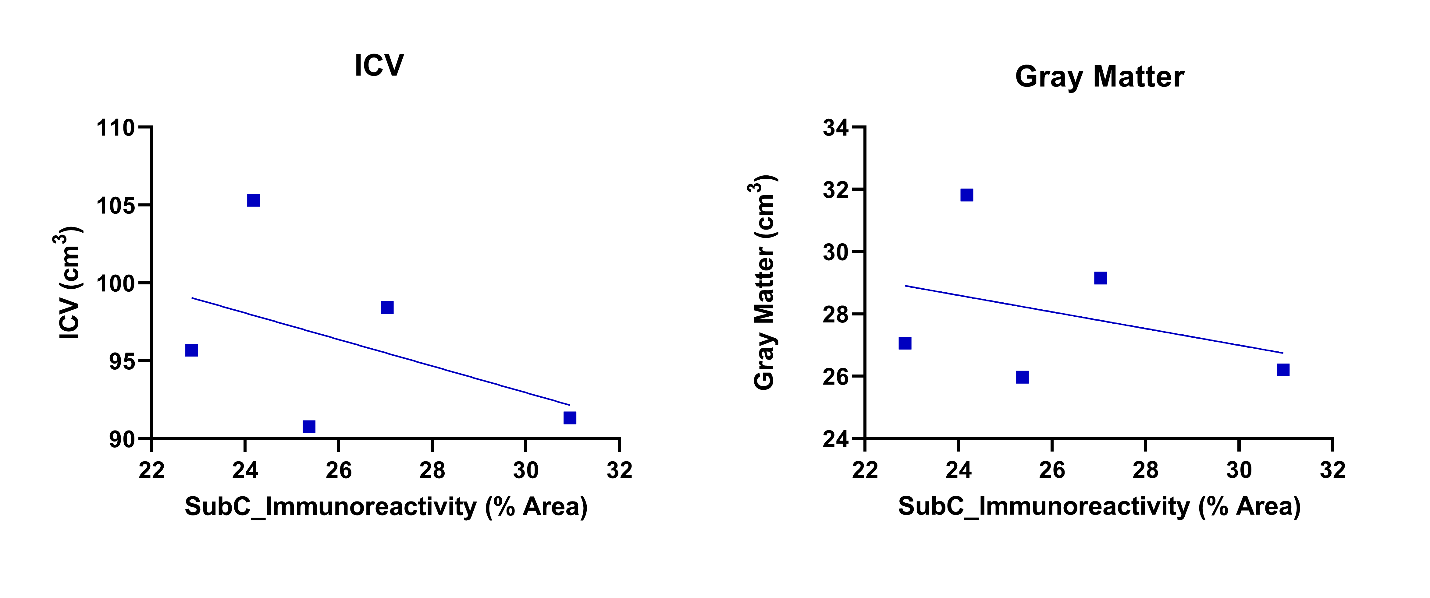


**Supplementary Figure 1:** For 17-month old *CLN2^R208X/R208X^*miniswine, a weak negative trend exists between the subunit C immunoreactivity area in the somatosensory cortex and the MRI brain volumetry measurement of the ICV and gray matter (Spearman correlation, ns)
